# A convolutional neural network to identify mosquito species (Diptera: Culicidae) of the genus *Aedes* by wing images

**DOI:** 10.1038/s41598-024-53631-x

**Published:** 2024-02-07

**Authors:** Felix G. Sauer, Moritz Werny, Kristopher Nolte, Carmen Villacañas de Castro, Norbert Becker, Ellen Kiel, Renke Lühken

**Affiliations:** 1 Bernhard Nocht Institute for Tropical Medicine, Hamburg, Germany; 2 The Deep Bench GmbH, Munich, Germany; 3 Faculty of Life Sciences, HAW Hamburg, Hamburg, Germany; 4 Carl von Ossietzky University, Oldenburg, Germany; 5 Faculty of Biosciences, University Heidelberg, Im Neuenheimer Feld 230, 69120 Heidelberg, Germany; 6 Institute of Dipterology (IfD)/KABS, Georg-Peter-Süß-Str. 3, 67346 Speyer, Germany

**Keywords:** Computational biology and bioinformatics, Classification and taxonomy

## Abstract

Accurate species identification is crucial to assess the medical relevance of a mosquito specimen, but requires intensive experience of the observers and well-equipped laboratories. In this proof-of-concept study, we developed a convolutional neural network (CNN) to identify seven *Aedes* species by wing images, only. While previous studies used images of the whole mosquito body, the nearly two-dimensional wings may facilitate standardized image capture and reduce the complexity of the CNN implementation. Mosquitoes were sampled from different sites in Germany. Their wings were mounted and photographed with a professional stereomicroscope. The data set consisted of 1155 wing images from seven *Aedes* species as well as 554 wings from different non-*Aedes* mosquitoes. A CNN was trained to differentiate between *Aedes* and non-*Aedes* mosquitoes and to classify the seven *Aedes* species based on grayscale and RGB images. Image processing, data augmentation, training, validation and testing were conducted in python using deep-learning framework PyTorch. Our best-performing CNN configuration achieved a macro F1 score of 99% to discriminate *Aedes* from non-*Aedes* mosquito species*.* The mean macro F1 score to predict the *Aedes* species was 90% for grayscale images and 91% for RGB images. In conclusion, wing images are sufficient to identify mosquito species by CNNs.

## Introduction

Mosquitoes (Diptera: Culicidae) are the most important arthropod vector group, causing approximately 350 million human infections and 500 thousand deaths per year^1^. Worldwide, more than 3500 extant mosquito species are registered^2^. The medical relevance of the species varies greatly, as each mosquito species is characterized by a species-specific vector capacity, e.g. distribution, breeding site types, host preferences or vector competence. Therefore, correct species identification is a crucial prerequisite to assess the local risk for mosquito-borne disease outbreaks and to implement appropriate control measures. Mosquitoes are commonly identified by taxonomic keys based on different morphological characters^3^. The morphological identification requires considerable entomological experience. Image-based species identification by means of convolutional neural networks (CNNs) may represent a cost-effective and time-saving alternative. Several studies demonstrated that CNNs have a high potential to support the species identification of mosquitoes^4,5^, even including the differentiation of cryptic species, which cannot be differentiated by morphology^6^.

A CNN is a specific type of artificial neural networks, which are particularly well suited for analysing visual data. Its development was inspired by the neural information processing of the visual cortex^7^, which is characterized by cortical areas that are specialized to certain aspects of visual perception, e.g. shape, colour or movement. Similar to the cortical areas, each convolution layer extracts certain features of the input images. A detailed explanation of its operating principle is given by Rawat and Wang^8^. Briefly, a CNN, when implemented as a supervised learning technique, uses a training data set of prior classified images. The CNN automatically learns to extract relevant features out of images and to classify the thereby resulting lower-dimensionality representation of the image by adjusting so called “neurons”. The neurons in a CNN are functionally similar to the synapses in the biological nervous system. During training, the CNN updates the biases of its neurons to minimize the prediction error^9,10^. Subsequently, a data set of out-of-sample (never-seen) images is used to test the classification capability of the trained network.

CNNs were demonstrated to have a high potential to support the accurate identification across various taxonomic groups, e.g. plants^11^, carabids^12^, chironomids^13^ or bumble bee species^14^. Likewise, CNN studies on mosquito identification showed promising results with an accuracy of up to 97%^4–6,15–19^. These studies trained their CNN with images from the whole mosquito body. At first glance, the usage of images from the whole mosquito body seems to be the straight-forward approach. However, image input has to be selected with caution, since the conditions of the photographed specimens can strongly influence the classification accuracy. For example, Couret et al.^6^ demonstrated that a CNN can distinguish between dried and frozen mosquito specimens, indicating that differences in the storage methods of the specimens could lead to a biased CNN training. In addition, the fragile and slender mosquito body is often damaged in field-sampled specimens and the three-dimensional body shape can complicate standardized image acquisition. The CNN requires images from different points of view to provide reliable results, which further increases the effort to create the image dataset. Hence, the CNN training based on the whole mosquito bodies usually needs a high number of images and intensive data augmentation to optimize classification accuracy^15^. This is, in turn, associated with a complex CNN implementation, including the use of deep neural network architectures and methods like Transfer learning, i.e. deep pre-trained networks, which requires expensive hardware and long training time^6,20^.

In order to overcome the described potential problems with images of the whole mosquito body, we here focussed on the use of mosquito wings for CNN-based species identification. Since wing beat frequency influence the assortative mating behaviour of mosquitoes, wings are considered to be under particular evolutionary selection pressure leading to species-specific wing morphology^21,22^. Studies analysing the wing shape by geometric morphometrics confirmed wings as sufficient anatomical feature to differentiate mosquito species^23,24^ including cryptic mosquito species^25^. For CNN-based species identification, the use of wing images provides certain advantages. The near two-dimensionality simplifies the capture of standardized images, reducing the image variety necessary for a robust CNN training. Thereby, one wing would allow species identification, even if the remaining body is damaged. Particularly, legs and scales are often damaged in field-sampled mosquitoes, while one well preserved wing is usually still available. In addition, mosquito wings can be easily mounted in an embedding medium and stably stored over a long period of time. This has a considerable advantage compared to the whole mosquito body, e.g. if interested in the integration of historic material. However, despite these potential advantages, we found no CNN-study focussing on wings for the identification of mosquito species.

Herein, we conducted a proof-of-concept study to analyse the potential of a CNN to identify mosquito species based on wing images. While previous studies with images from the whole mosquito body used deep pre-trained neural networks, we developed a CNN with a rather shallow architecture and therefore lower hardware requirements. Two CNNs of the same architecture were trained, differing only in the number of neurons of the last fully connected layer. The first was trained to differentiate between *Aedes* and non-*Aedes* mosquitoes and the second to distinguish seven *Aedes* species, including the exotic *Aedes albopictus* (Skuse, 1894) and the native species *Aedes communis* (De Geer, 1776), *Aedes cinereus* Meigen, 1818, *Aedes punctor* (Kirby, 1837), *Aedes rusticus* (Rossi, 1790), *Aedes sticticus* (Meigen, 1838) and *Aedes vexans* (Meigen, 1830) collected from different sites in Germany. We trained the CNNs to differentiate the classes based on RGB and grayscale images, respectively. We hypothesized that grayscale images may not result in the loss of important information for species identification, since wing scales of native mosquitoes are pale or black and never colourful. In addition, the conversion of grayscale images increases the contrasts between wing veins and background. At the same time, it reduces the file size per image, and thus the computing effort and the requirements for the CNN complexity.

*Aedes* is the most divers genus in Central Europe^26^. Females of the genus are difficult to identify by classical taxonomic keys, since their differentiation predominantly relies on scale patterns, which are fragile and often damaged in field-sampled mosquitoes^2^. This underlines the demand on complementary tools for fast and reliable species identification of *Aedes* species.

## Material and methods

### Data collection

The study was based on 1,155 wing photos from female *Aedes* specimens, including 165 *Ae. albopictus*, 165 *Ae. cinereus*, 165 *Ae. communis*, 165 *Ae. punctor*, 165 *Ae. rusticus*, 165 *Ae. sticticus* and 165 *Ae. vexans*. As unknown-class we integrated further 554 wing photos from common non-*Aedes* mosquito species in Germany, including 61 *Anopheles claviger* (Meigen, 1804), 196 *Anopheles maculipennis* s.l., 11 *Anopheles plumbeus* Stephens, 1828, 214 *Culex pipiens* s.s./*Cx. torrentium* and 72 *Coquillettidia richiardii* (Ficalbi, 1889). The native mosquito species originated from at least three different sampling locations for each species and were collected with carbon dioxide baited BG sentinel traps (Biogents, Regensburg, Germany). The field-sampled mosquitoes were directly killed and stored at − 20 °C until further preparation. All specimens were identified by morphology^3,27^. After the morphological species identification, the right wing of each specimen was removed and mounted with euparal (Carl Roth, Karlsruhe, Germany) on microscopic slides. Subsequently, the mounted wings were photographed with a stereomicroscope (Leica M205 C, Leica Microsystems, Wetzlar, Germany) under 20 × magnification using standardized illumination under and exposure time (279 ms). A CNN uses any distinctive character in the images to optimize for more accurate class predictions. For example, differences in the illumination of the background between the images of the different species could be exploited by the CNN during training. This would lead to predictions that seem correct for the specific data set, but are based on irrelevant, non-generalized features, leading to a reduced transferability of the network predictions. Therefore, we used dome illumination (MEB 111, Leica Microsystems, Wetzlar, Germany), which largely shields the samples from ambient light during image collection.

### Data split for training, validation and testing

Before training, the data sets were randomly split into data for training (75% of the images), for validation (15% of the images) and for testing (15% of the images). Thereby, the validation images are used as in-training reference to track classification accuracy during training process, while the testing images are out-of-training and will only be used to check the accuracy of the CNNs after the training progress is completed. The macro-average metrics precision, recall (i.e. sensitivity), and F1 scores were calculated to quantify the testing results for each CNN training.

### Image pre-processing and data augmentation

Wing photos were cropped in the centre and resized to 256 × 256 pixels before being used in the CNN. Each image of training data sets was augmented 32 times. We used a randomized rotation in the range of − 15° to + 15° as well as a randomized horizontal and vertical shift in a range of − 20 to + 20% to generate random alterations of the original images. Moreover, we incorporated zoom and crop of the input images as additional augmentation techniques. The maximum values for the zoom and crop augmentation were identified in a preliminary test. Thereby, we systematically evaluated the CNN performance across a range of maximum zoom levels from 1 × and 2 × and for an image crop between 10 to 90%. The best performance was reached with a maximum zoom of 1.5 × and a crop limit of 40% of the resized input images. Data augmentation increases and generalizes the pool of training samples. It is commonly applied, when dealing with rather small data sets for a CNN training and was already demonstrated to distinctly increase the final accuracy in entomological studies on species identification^5,6,13,15^.

### CNN configuration and training

**Figure 1:**
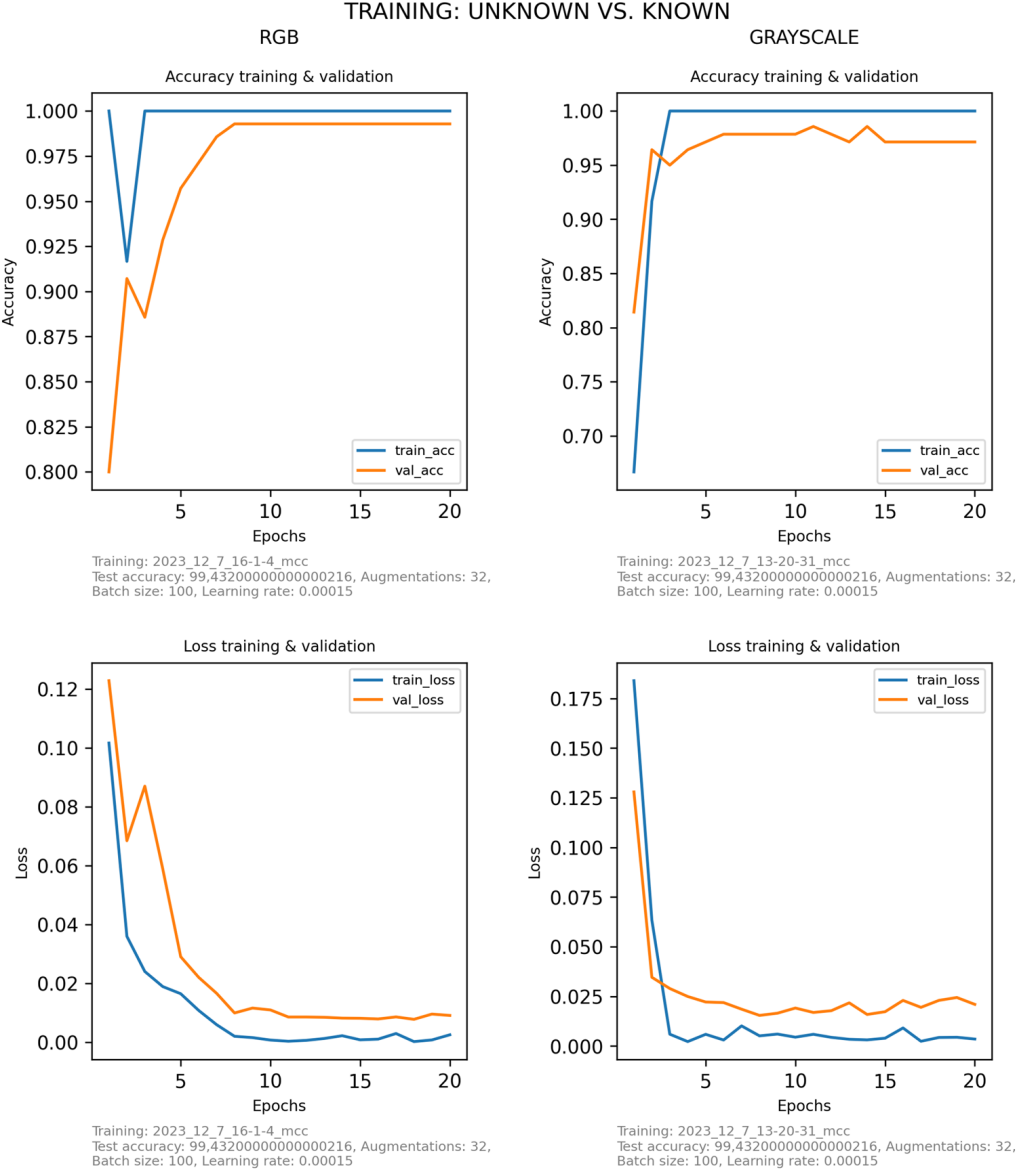
Evolution of the accuracy and loss during the training (blue) and validation (orange) process along the epochs. The figures refer to the best-performing CNN model to distinguish *Aedes* and non-*Aedes* mosquitoes based on RGB (left) or grayscale (right) images.

**Figure 2:**
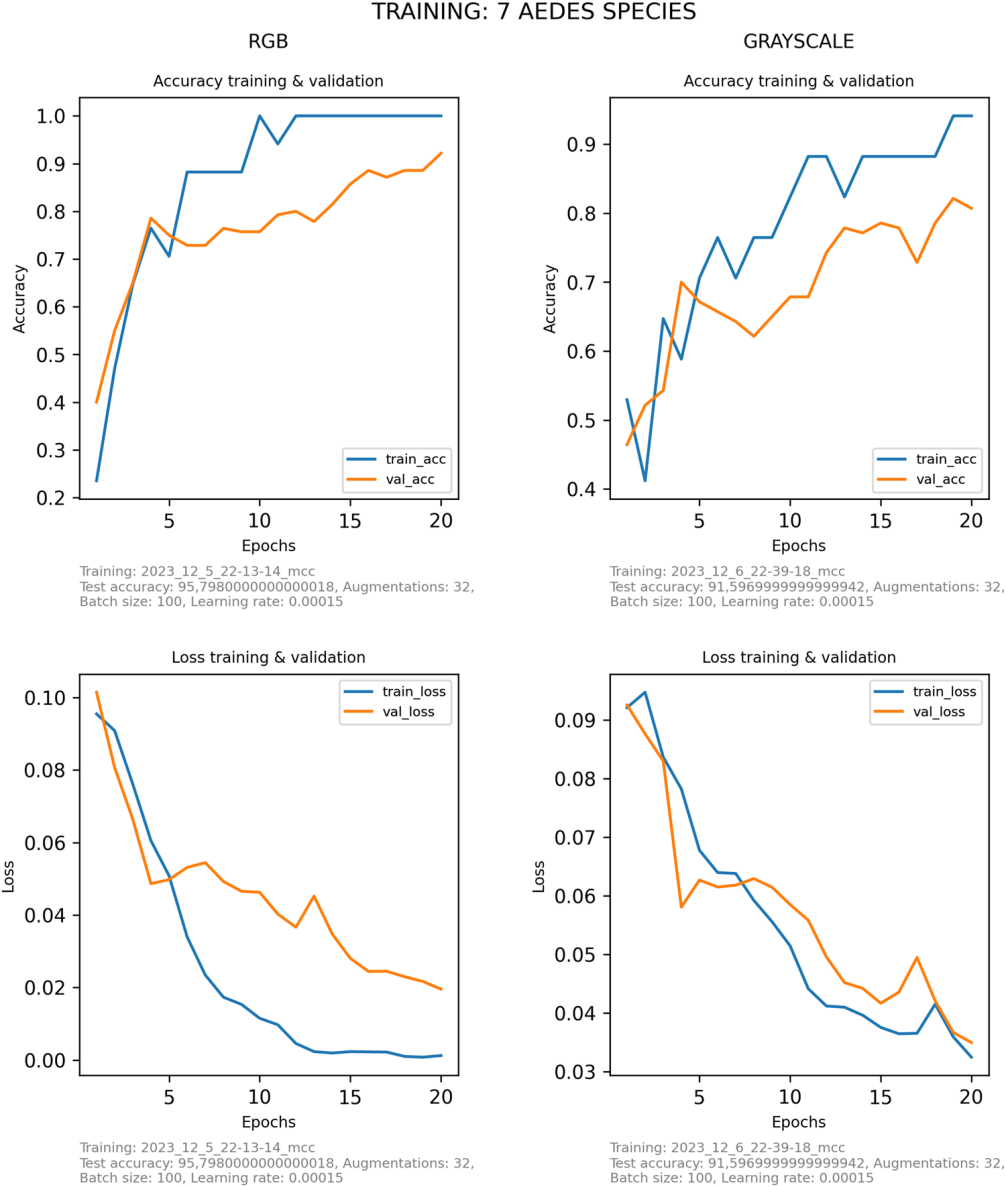
Evolution of the accuracy and loss during the training (blue) and validation (orange) process along the epochs. The figures refer to the best-performing CNN model to distinguish the seven *Aedes* species based on RGB (left) or grayscale (right) images.

Several iterations with different input image sizes and CNN-architectures were tested to find a compromise between level of image resolution, computation expense and classification accuracy. The final CNN used input images with 256 × 256 pixels and consisted of four convolutional layers and one fully-connected layer. For the different trials, we used the same CNN architecture. The only changes implemented in the CNN for RGB images are adjustments allowing to work with three-channel input. The difference between the CNN to differentiate *Aedes* from non-*Aedes* and the seven *Aedes* species is the last fully connected layer, either consisting of two or seven neurons. The rather shallow architecture was chosen to match the small sample size and to avoid extensive computing time during training. After the basic CNN architecture was set, the learning parameters were fine-tuned by trial and error. The setting of the hyperparameters is a crucial step, as it can strongly influence training time and prediction performance. There exists no general gold standard in the adjustment of hyperparameters. Instead, these parameters must be optimized for each dataset and CNN architecture^28^. During hyperparameter-tuning, we optimized the number of epochs, the batch size and the learning rate. Number of epochs controls how often the CNN goes through the in-sample training data. The training accuracy commonly reaches an optimum after a certain number of epochs. Further epochs can lead to over-fitted models and unnecessarily increases the computing time. In this study, we used 20 epochs as a compromise between training time and performance (Figs. 1 and 2).

Batch size defines the number of images after the CNN updates its learning process. After each batch, the algorithm calculates a misclassification error and updates its model to optimize training accuracy. Batching reduces computing time, as the samples in one batch can be processed in parallel. In addition, it avoids adjusting of the CNN to individual samples rather than to the entirety of the data set, which otherwise could lead to biased interpretations based on individual samples. Our best-performing trainings used a batch size of 100. Learning rate defines how strong the CNN responds to misidentifications after each batch. A high learning rate can result in unstable training, since the CNN overcompensate errors of the preceding batch, whereby a too small learning rate prolong or even negate the optimisation process. The optimal learning rate in our most successful training session was 0.00015. We utilized the Adam optimizers for model training^29^. To address overfitting during CNN training, weight decay regularization with a coefficient of 0.0005 was employed. In order to check the consistency of the accuracy results, the training with the most successful CNN configuration was repeated and tested four times for each set-up, i.e. using grayscale or RGB images to differentiate *Aedes* from non-*Aedes* and to differentiate the seven *Aedes* species, respectively.

### Guided gradient-weighted class activation mapping

Guided Gradient-weighted Class Activation Mapping (Grad-CAM) was applied to investigate the underlying cause for the decision-making of the CNN^30^. Guided Grad-CAM helps to get insights into the decision-making process and to detect misbehaviour of neural networks, by visualising image regions that are decisive for a classification. Therefore, it combines two components, Grad-CAM and Guided Backpropagation. Briefly, Grad-CAM aims to visualize class-discriminative image regions by assigning importance values to each neuron in a network layer for a certain decision^30^. It is usually applied to the last convolutional layer, creating a low-resolution output. It is a compromise between high-level semantics and spatial information, but it is impossible to match importance values of neurons to specific pixels in the input image^30^. Guided Backpropagation can then further increase the resolution of the visualization of discriminate image regions. It computes the gradient of the target class with respect to the input image by considering only non-negative neuron activations and can thus capture pixels in the input image detected by the neurons of the last convolutional layer. When testing the accuracy of the trained CNNs, Guided Grad-CAM was applied to the last (4^th^) convolutional layer for each image of the out-of-sample data. For visualization, the resulting heat maps, showing the discriminative image regions, were superimposed with the original wing images. The consistency of the heat map patterns was inspected visually.

### Hardware and programming language

The programming was done in Python (version 3.9.6). For the neural network aspects, the deep learning framework Pytorch (version 1.10.2)^31^ was used, while image pre- and post-processing as well as visualization were done with the Python libraries OpenCV2 (version 3.4.5) and Matplotlib (version 3.5.1). All calculations were conducted with a notebook (Intel Core 7-6700HQ 260 GHz, 16 GB RAM) on a graphic card (NIVIDIA GeForce GTX 1060). The computing time was approximately 80 min when using RGB images and approximately 60 min when using grayscale images (Supplementary Material S1: log file).

## Results

**Figure 3:**
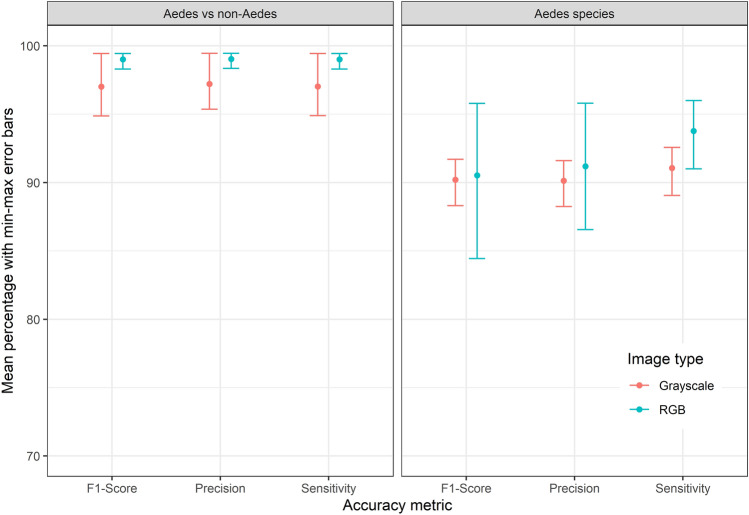
Mean macro-averaged metrics (F1-score, precision and sensitivity) with maximum and minimum values of the four CNN trainings conducted with grayscale (red) and RGB (blue) images to differentiate the seven *Aedes* species (left) and *Aedes* from non-*Aedes* species (right), respectively.

**Figure 4:**
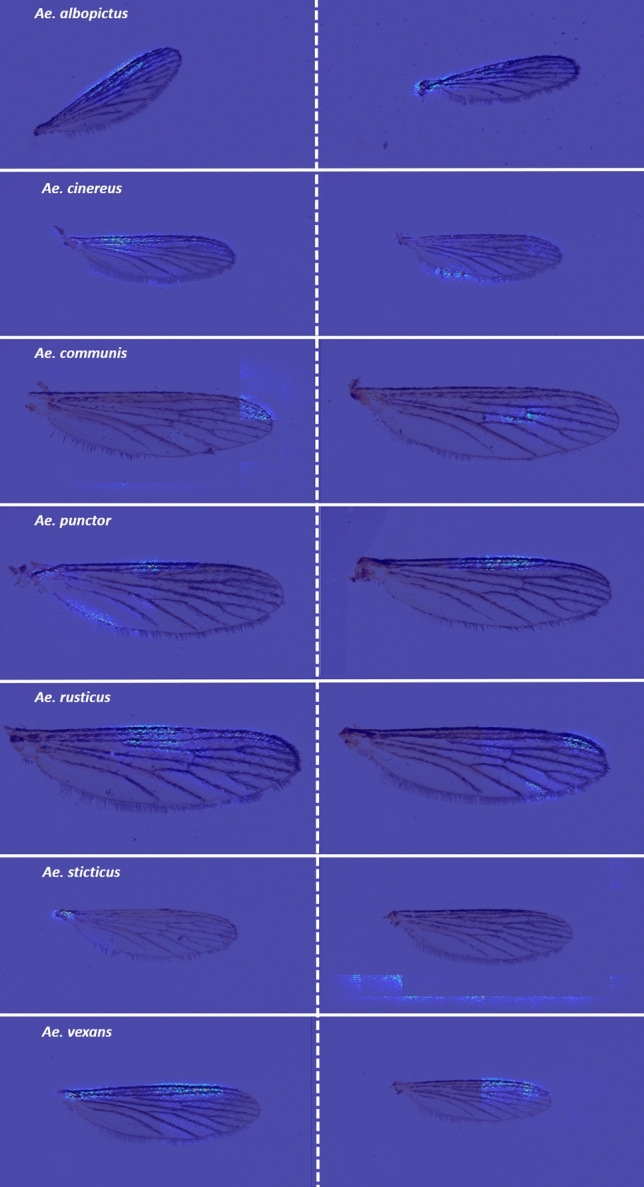
Each row shows exemplary pictures per *Aedes* species of the Guided Grad-Cam output based on the CNN training with the highest accuracy. The bright image regions indicate areas most relevant for species discrimination.

For the differentiation between *Aedes* and non-*Aedes* species based on grayscale images, the mean macro-averaged scores were 97% (min–max: 95–99%) for the precision, 97% (95–99%) for the sensitivity and 97% (95–99%) for the F1-score (Fig. 3). The differentiation between *Aedes* and non-*Aedes* species based on RGB images achieved 99% (98–99%), for the precision, sensitivity and F1-score, respectively (Fig. 3). For the differentiation of the seven *Aedes* species, the mean scores based on grayscale images were 90% (88–92%), 91% (89–93%) and 90% (88–92%) for the precision, sensitivity and F1-score, respectively (Fig. 3). When using RGB images to differentiate the seven *Aedes* species, the precision achieved 91% (87–96%), the sensitivity was 94% (91–96%) and F1-score was 91% (84–96%) (Fig. 3). *Aedes albopictus* was detected with an accuracy of 100% for both grayscale and RGB images (Tables 1 and 2). Misidentifications were only found among the native *Aedes* species, particularly between *Ae. communis* and *Ae. punctor* (Tables 1 and 2). The most relevant image areas for decision making were visualised by Guided Grad-CAM. The resulting pictures indicated that the neurons were usually most active on the wing veins (Fig. 4).

**Table 1:** Confusion matrix showing the testing results for the CNN trainings based on grayscale images.

	*Ae. albopictus*				*Ae. cinereus*				*Ae. communis*				*Ae. punctor*				*Ae. rusticus*				*Ae. sticticus*				*Ae. vexans*			
	R1	R2	R3	R4	R1	R2	R3	R4	R1	R2	R3	R4	R1	R2	R3	R4	R1	R2	R3	R4	R1	R2	R3	R4	R1	R2	R3	R4
*Ae. albopictus*	**17**	**17**	**17**	**17**	0	0	0	0	0	0	0	0	0	0	0	0	0	0	0	0	0	0	0	0	0	0	0	0
*Ae. cinereus*	0	0	0	0	**17**	**17**	**17**	**17**	0	0	0	0	0	0	0	0	0	0	0	0	0	0	0	0	0	0	0	0
*Ae. communis*	0	0	0	0	0	0	0	0	**13**	**13**	**14**	**14**	*2*	0	*2*	*2*	*1*	*1*	*1*	*1*	0	0	0	0	0	0	0	0
*Ae. punctor*	0	0	0	0	0	0	0	0	*3*	*4*	*3*	*3*	**15**	**17**	**15**	**15**	0	0	0	0	0	0	0	0	*1*	*2*	*1*	*2*
*Ae. rusticus*	0	0	0	0	0	0	0	0	0	0	0	0	0	0	0	0	**16**	**16**	**16**	**16**	0	0	0	0	0	0	0	0
*Ae. sticticus*	0	0	0	0	0	0	0	0	0	0	0	0	0	0	0	0	0	0	0	0	**12**	**15**	**14**	**12**	0	*1*	0	*1*
*Ae. vexans*	0	0	0	0	0	0	0	0	*1*	0	0	0	0	0	0	0	0	0	0	0	*5*	*2*	*3*	*5*	**16**	**14**	**16**	**14**
Accuracy in %	100	100	100	100	100	100	100	100	76	76	82	82	88	100	88	88	94	94	94	94	71	88	82	71	94	82	94	82

The training was repeated in four runs (R). Bold highlights correct classification. Italic cells highlight misclassified specimens.

**Table 2:** Confusion matrix showing the testing results for the CNN trainings based on RGB images.

	*Ae. albopictus*				*Ae. cinereus*				*Ae. communis*				*Ae. punctor*				*Ae. rusticus*				*Ae. sticticus*				*Ae. vexans*			
	R1	R2	R3	R4	R1	R2	R3	R4	R1	R2	R3	R4	R1	R2	R3	R4	R1	R2	R3	R4	R1	R2	R3	R4	R1	R2	R3	R4
*Ae. albopictus*	**17**	**17**	**17**	**17**	0	0	0	0	0	0	0	0	0	0	0	0	0	0	0	0	0	*1*	0	0	0	0	0	0
*Ae. cinereus*	0	0	0	0	**17**	**17**	**17**	**17**	0	0	0	0	0	0	0	0	0	0	0	0	0	0	0	0	0	0	0	0
*Ae. communis*	0	0	0	0	0	0	0	0	**17**	**17**	**16**	**17**	*5*	*12*	*3*	*10*	*1*	0	0	*1*	0	0	0	*1*	0	0	0	0
*Ae. punctor*	0	0	0	0	0	0	0	0	0	0	*1*	0	**12**	**4**	**14**	**7**	0	0	0	0	0	0	0	0	0	0	0	0
*Ae. rusticus*	0	0	0	0	0	0	0	0	0	0	0	0	0	*1*	0	0	**16**	**17**	**17**	**16**	0	0	0	0	0	0	0	0
*Ae. sticticus*	0	0	0	0	0	0	0	0	0	0	0	0	0	0	0	0	0	0	0	0	**15**	**14**	**17**	**15**	0	0	*1*	0
*Ae. vexans*	0	0	0	0	0	0	0	0	0	0	0	0	0	0	0	0	0	0	0	0	*2*	*2*	0	*1*	**17**	**17**	**16**	**17**
Accuracy in %	100	100	100	100	100	100	100	100	100	100	94	100	71	24	82	41	94	100	100	94	88	82	100	88	100	100	94	100

The training was repeated in four runs (R). Bold highlights correct classification. Italic cells highlight misclassified specimens.

## Discussion

In this study, we developed a CNN to differentiate mosquito species by wing images. Previous CNN studies used images from the whole mosquito body from varying postures and different image quality^5,32^. Motta et al.^32^ even included images from a mobile phone camera. Using images from the whole mosquito body is less time-consuming, but the CNN implementation is more labour-intensive, as the higher complexity of the images require deeper neural network architectures and methods like transferred learning, i.e. the use of deep pre-trained networks, which requires more expensive hardware and longer training time^6,20^. In comparison, wing images need some lab preparation, but images of the mounted wings are much easier to standardize. This favours the use of a relatively simple CNN architecture and can reduce the image variety, i.e. less images from different postures, and reduce the quantity of images necessary for a robust CNN training. Motta et al.^32^ and Park et al.^5^ reported a validation accuracy of 75% and 97%, respectively. From an entomological point of view, the chosen species/taxa *Ae. aegypti*, *Ae. albopictus* and *Cx. quinquefasciatus* by Motta et al.^32^ and *Ae. albopictus, Ae. vexans, Anopheles* spp., *Cx. pipiens* and *Cx. tritaeniorhynchus* by Park et al.^5^ are taxonomically distant. The seven *Aedes* species in the present study are more closely related and at least partly more difficult to discriminate by morphology. However, no misclassification was found for the invasive *Ae. albopictus*. Misclassified individuals were merely observed between the more closely related native *Aedes* species. This is, in turn, a further indication that the CNN actually learned morphological wing patterns for species identification as a close evolutionary relationship is often reflected in a similar wing geometry^23,33^. The output of the guided Grad-CAM supports this assumption, as the neurons were usually most active on the wing veins. Similarly, the wing vein patterns are also a relevant anatomical wing feature for entomologists to identify mosquito species^3^.

Lowest accuracies were obtained for *Ae. punctor* and *Ae. communis*, which were misclassified with each other. These two species are very similar to each other. They are commonly distinguished by the postprocoxal scales at the thorax, i.e. a small patch of scales, which is present in *Ae. punctor* and absent in *Ae. communis*^3,27^. Wing patterns are not described as important morphological characters^3,27^ and a study using landmark-based geometric wing morphometrics showed a rather low accuracy (app. 75% for the differentiation of *Ae. communis* and *Ae. punctor*)^23^. Similarly, the CNN had a lower accuracy for the closely related *Ae. sticticus* and *Ae. vexans*. Hence, the low CNN accuracy to distinguish the two species pairs probably reflect their close morphological and phylogenetic relationship.

As demonstrated previously, high quality body images could be used to distinguish cryptic *Anopheles*^6^ and cryptic *Culex*^19^ species via CNN. Thereby, Couret, et al.^6^ also showed that the storage method of the mosquitoes (here: flash freezing vs. dried) can influence the network training. This might be less problematic for wing images, which can be easily stored in an embedding medium for permanent storage. Thus, future research should analyse the potential of CNN to distinguish cryptic species by means of wing images.

The current CNN training relies on a rather low number of images, e.g. the widely used ImageNet data set provides at least 500 images per class^34^. While the number of samples was sufficient for a first case study, it is expected that the CNN performance will increase with a larger amount of training data. Further wing images would probably increase the accuracy and robustness of the developed CNN. In addition, it would allow a greater variety within the image quality. This should also include different camera systems to enhance the practical orientation, e.g. mobile phone camera. Moreover, a systematic comparison of the performance of different CNNs based on the same datasets including a comparison of mosquito bodies and wings would be desirable. Both image types might have specific advantages for the CNN performance. The use of mosquito wings might also complement future CNNs, if the mosquito body alone is not sufficient for an accurate species identification. The increasing amount of available data in combination with an increase in computing capacity will probably improve the performance of future CNNs and can complement the mosquito species identification. However, data input must be chosen carefully. The quality of training remains dependent on the correct classification by entomologists, since misidentified species lead to wrongly trained neural networks.

### Supplementary Information

Supplementary Table S1.

## Data Availability

Supplementary Material S1: log file. The source codes will be provided through the GitHub repository: https://github.com/mwdevhub/Mosquito_Species_Classification_CNN. All wing images are provided via Dryad: 10.5061/dryad.vx0k6djz9.
